# Supplementary material for: An algorithmic approach to detect generalization in sketch maps from sketch map alignment
Source: PLoS One. 2024 Jun 26;19(6):e0304696. doi: 10.1371/journal.pone.0304696 (PMC11206956; doi:10.1371/journal.pone.0304696)
Supplement: Attachment — Submitted filename: changeReport.docx [file pone.0304696.s001.docx]

Dear Reviewers,

Thank you for your valuable comments. It has helped us to improve the quality of the paper significantly. Below you can find the reply addressed to each of the suggestions.

Reviewer #1: Andrzej Chybicki, PhD Eng.

The manuscript's length is excessive, leading to reader confusion. A more concise presentation, especially regarding generalization approaches, would enhance readability. These approaches, already discussed in prior studies, could be summarized briefly.

The length of the manuscript has been reduced by four pages.

There is noticeable repetition within the text. For example, the content description of Figure 1 is redundant on pages 6 and 8.

The explanation of Figure 1 on page 8 has been removed.

Specific concerns and suggestions include:

1) The detailed description of generalization techniques (e.g., on page 20) is overly extensive. A brief summary with references to existing literature would suffice.

The description of generalization technique in section 2.2 has been moved to fit within Figure 2. The description on page 20 is however not the explanation of generalization type, but rather how the algorithm detects the different generalization types. We apologize for not being clear with it. The texts on page 20 have been slightly modified to make it clear that the texts are description of generalization algorithm and not of generalization operator.

2) The method for identifying regions of interest from sketch drawings needs clarification. It's unclear whether this requires manual input or is automatically detected. In other words, how do you specify OSM map region based from sketch map?

It requires manual input. We have mentioned it now in section 3.3.

3) The computational complexity of the proposed method is not addressed. An analytical discussion of its feasibility for large datasets (1000+ features) would be beneficial.

We have added the aspect of computational comlexity in terms of time and storage in section 4.2

4) My biggest concern is that the Table 2's content and the abbreviation 'SM' are unclear.

Furthermore, there are repetitive row names, such as 'SMO 0 - Amalgamation,' which hinder the interpretation of results.

We have modified the Table 2’s content to make it clear and also added explanation on Table 2’s content in section 4.1.

5) Chapter 5 appears to add little value. Consider removing or integrating it with Section 6 for brevity and clarity.

We have removed chapter 5 and integrated it with the conclusion in section 5 (previously section 6)

Overall, the study is a valuable contribution and seems fit for publication upon revision. I recommend focusing on Section 4, elaborating on the results and providing a more detailed discussion. A minor note: mentioning raster generalization may be unnecessary, as sketch maps inherently involve vector data.

We have removed the texts on raster generalization (section 2.3)

Reviewer #2: An Algorithmic Approach to Detect Generalization in Sketch Maps from Sketch Map Alignment

This is an interesting topic. The idea and contributions of this study appear to be a valid one, and together with their earlier work, it has the potential to offer a helpful guide for various disciplines. On the whole, the paper is structured reasonably, and its intentions and argument are relatively clear.

The discussion and conclusion sections are potentially the most interesting parts of the paper, and at present the least well-developed. A helpful way forward would be to identify a hierarchy to structure the implication more effectively.

Thanks for pointing this out. We have elaborated the discussion sections and conclusion sections. The implication has been made clear in the conclusion.

It would be better for a new iteration of this paper to highlight the similarities and differences of this research compared to previous studies such as the following, and to specifically locate new concepts in the form of a review analysis.
Finally, major revision is suggested.

- Hadlos, A., Opdyke, A., & Hadigheh, S. A. (2022). Where does local and indigenous knowledge in disaster risk reduction go from here? A systematic literature review. International Journal of Disaster Risk Reduction, 103160.

- Membele, G. M., Naidu, M., & Mutanga, O. (2022). Examining flood vulnerability mapping approaches in developing countries: A scoping review. International Journal of Disaster Risk Reduction, 69, 102766.

- Klonner, C., Hartmann, M., Dischl, R., Djami, L., Anderson, L., Raifer, M., ... & Porto de Albuquerque, J. (2021). The sketch map tool facilitates the assessment of OpenStreetMap data for participatory mapping. ISPRS International Journal of Geo-Information, 10(3), 130.

- Shen, Y., Ai, T., Li, J., Huang, L., & Li, W. (2020). A progressive method for the collapse of river representation considering geographical characteristics. International Journal of Digital Earth, 13(12), 1366-1390.

- Jakobi, Á., & Pődör, A. (2020). GIS-based statistical analysis of detecting fear of crime with digital sketch maps: A Hungarian multicity study. ISPRS International Journal of Geo-Information, 9(4), 229.

- Brandt, K., Graham, L., Hawthorne, T., Jeanty, J., Burkholder, B., Munisteri, C., & Visaggi, C. (2020). Integrating sketch mapping and hot spot analysis to enhance capacity for community‐level flood and disaster risk management. The Geographical Journal, 186(2), 198-212.

Thank you for the references. We have discussed how our approach of handling generalization in sketch map can enhance the previous works in section 2.1. We have integrated all your suggested references into the text.
